# Network-assisted analysis of GWAS data identifies a functionally-relevant gene module for childhood-onset asthma

**DOI:** 10.1038/s41598-017-01058-y

**Published:** 2017-04-20

**Authors:** Y. Liu, M. Brossard, C. Sarnowski, A. Vaysse, M. Moffatt, P. Margaritte-Jeannin, F. Llinares-López, M. H. Dizier, M. Lathrop, W. Cookson, E. Bouzigon, F. Demenais

**Affiliations:** 1 INSERM, Genetic Variation and Human Diseases Unit, UMR-946, Paris, France; 2 Université Paris Diderot, Université Sorbonne Paris Cité, Institut Universitaire d’Hématologie, Paris, France; 3 Genomic Medicine Section, National Heart Lung Institute, Imperial College London, London, UK; 4 Machine Learning and Computational Biology Lab, Department of Biosystems Science and Engineering, ETH Zürich, Basel, Switzerland; 5 McGill University and Genome Québec Innovation Centre, Montréal, Québec Canada

## Abstract

The number of genetic factors associated with asthma remains limited. To identify new genes with an undetected individual effect but collectively influencing asthma risk, we conducted a network-assisted analysis that integrates outcomes of genome-wide association studies (GWAS) and protein-protein interaction networks. We used two GWAS datasets, each consisting of the results of a meta-analysis of nine childhood-onset asthma GWASs (5,924 and 6,043 subjects, respectively). We developed a novel method to compute gene-level *P*-values (fastCGP), and proposed a parallel dense-module search and cross-selection strategy to identify an asthma-associated gene module. We identified a module of 91 genes with a significant joint effect on childhood-onset asthma (*P* < 10^−5^). This module contained a core subnetwork including genes at known asthma loci and five peripheral subnetworks including relevant candidates. Notably, the core genes were connected to *APP* (encoding amyloid beta precursor protein), a major player in Alzheimer’s disease that is known to have immune and inflammatory components. Functional analysis of the module genes revealed four gene clusters involved in innate and adaptive immunity, chemotaxis, cell-adhesion and transcription regulation, which are biologically meaningful processes that may underlie asthma risk. Our findings provide important clues for future research into asthma aetiology.

## Introduction

Asthma is a common chronic inflammatory disease of the airways, characterized by varying age at onset and clinical presentation^1^. It is currently estimated that 334 million people suffer from asthma worldwide and 14% of the world’s children experience asthma symptoms^1^. Although environmental factors play an important role in asthma, estimates of heritability of asthma range from 35% to 75%^2^, which suggests significant genetic contribution. There have been considerable efforts to characterize the genetic factors underlying asthma, including candidate gene studies, positional cloning studies and more recently genome-wide association studies (GWAS)^3, 4^. Although these studies have been successful in identifying novel loci, the genetic factors identified to date explain only a small part of asthma risk. Moreover, heterogeneity is a hallmark of asthma. Genetic heterogeneity according to age of onset of asthma has been evidenced, with genetic factors appearing to play a more important role in childhood-onset asthma^5, 6^.

Typically, GWAS focus on testing association of disease with individual SNPs over the genome and only top-ranked SNPs with strongest statistical evidence for association are reported. GWAS are therefore underpowered to detect genetic variants which have small marginal effect but rather act jointly or interact with each other in disease or trait variability. To complement the typical single-marker analysis, more sophisticated analyses of GWAS data, which integrate biological knowledge with GWAS outcomes, have been proposed to allow detecting sets of functionally related genes that jointly affect disease risk. Among many of these approaches stands the network-assisted analysis that integrates GWAS results with protein-protein interaction (PPI) network to identify gene modules (subnetworks) enriched in association signals. The rationale behind it is the principle of “guilt-by-association”, which states connected genes (or gene products) are usually participating in the same, or related, cellular functions^7, 8^. Therefore, network-assisted analysis is a promising approach to discover functionally related genes that have a small marginal effect but rather act jointly in disease susceptibility.

In spite of such advantages, network-assisted analysis is also facing challenges. In a classical GWAS, association tests are typically performed at the SNP level, yet the basic entity of PPI network is gene products (proteins). An essential question is how to aggregate signals at SNP-level into gene-level. A popular approach is to take the best SNP from all SNPs mapped to a gene as gene-level *P-*value. However, longer genes represented by more SNPs are more likely to have small *P-*values by chance^9, 10^. Another challenge of network-assisted analysis is that most algorithms for searching gene modules are sensitive to the input data: the PPI network and GWAS results. It is well known that GWAS vary in their results for many reasons such as study design, genetic background of the populations, disease heterogeneity, and influence of environmental exposures or simply because of random variation. Therefore, appropriate network-analysis strategies need to be implemented to identify reliable disease-associated gene modules.

In the present study, we conducted a network-assisted analysis by integrating childhood-onset asthma (COA) GWAS results with experimentally verified human PPI network information to identify a set of interconnected genes that significantly contributes to COA risk. We used two large GWAS datasets which consisted of the results of meta-analyses of nine COA GWAS each (5,924 subjects for the first dataset, 6,043 subjects for the second dataset), that were part of the European Gabriel asthma consortium. To address the challenges mentioned above, we first developed an efficient method, named fastCGP, to compute gene-level *P-*values from GWAS SNP-level *P-*values. Then, we used a parallel dense-module search and cross-selection strategy to search for a consistent gene module between the two datasets. We identified a module of 91 genes significantly associated with childhood-onset asthma, including both known and novel candidate genes. Inspection of the interconnected components of this module together with functional enrichment analysis revealed biologically meaningful processes that underlie the risk of childhood-onset asthma.

## Results

**Figure 1:**
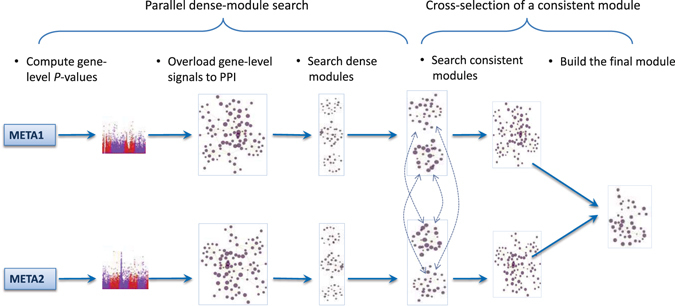
Workflow of the parallel dense-module search and cross-selection strategy. Individual SNP-level *P*-values from two independent childhood-onset asthma GWAS datasets (META1 and META2) were used as input for our network analysis. Gene-level *P*-values, computed from SNP-level *P*-values using fastCGP were converted to z-scores and overloaded to the PPI. The Dense Module Search algorithm was applied to each scored-PPI in parallel to search for dense modules. Modules with highest consistency between the two datasets were selected to build the final module.

The different steps of the proposed parallel dense-module search and cross-selection strategy are summarized in Fig. 1.

### Identification of a module enriched with childhood-onset asthma-associated genes

We used individual SNP-level *P-*values from two independent COA GWAS datasets as input data to perform network-assisted analysis. These two datasets were the results of meta-analyses of nine COA GWAS each (named META1 and META2, respectively) and included 2,370,689 unique SNPs. The two datasets that were meta-analysed were obtained by randomly splitting the total set of 18 Gabriel Consortium COA GWAS into two sets of similar size (3,031 cases and 2,893 controls in the first set; 2,679 cases and 3,364 controls in the second set).

We first combined SNP-level *P-*values into gene-level *P-*values using a novel gene-based method named fastCGP. fastCGP starts by mapping SNPs to genes (between the start site and 3′-untranslated region of each gene) using dbSNP Build 132 and human Genome Build 37.1, making a total of 24,120 genes with at least one SNP mapped. Then, gene-level *P-*values were taken as the best SNP *P-*values among all SNPs mapped to the gene and were corrected for the gene length bias using circular genomic permutation of SNP *P*-values, which allows taking into account the linkage disequilibrium (LD) between SNPs (see Methods and Supplementary Information for details). The resulting gene-level *P-*values in META1 and META2 are shown in Supplementary Fig. S1. Gene-level *P-*values were transformed to z-scores and overloaded to PPI, resulting in a scored-PPI for META1 and a scored-PPI for META2. The scored-PPIs consisted of 12,709 proteins and 123,608 interactions.

The Dense Module Search (DMS) algorithm^11^ was applied to each scored-PPI in parallel to identify modules enriched in association signals (quantified by a module score, which is the mean of z-scores of module genes). A total of 10,439 modules were generated in META1 and 10,429 modules in META2. As expected, because of the nature of DMS algorithm, there is considerable gene overlap between the modules generated in each dataset (Supplementary Fig. S2). We reduced such redundancy by hierarchically merging the raw modules within dataset until all pairwise module similarities were smaller than 0.5 (a similarity of *r* implies two modules share ~100*r*% of their genes), which resulted in 1,127 modules in META1 and 952 modules in META2. The overlap between the merged modules was largely reduced (Supplementary Fig. S2).

**Figure 2:**
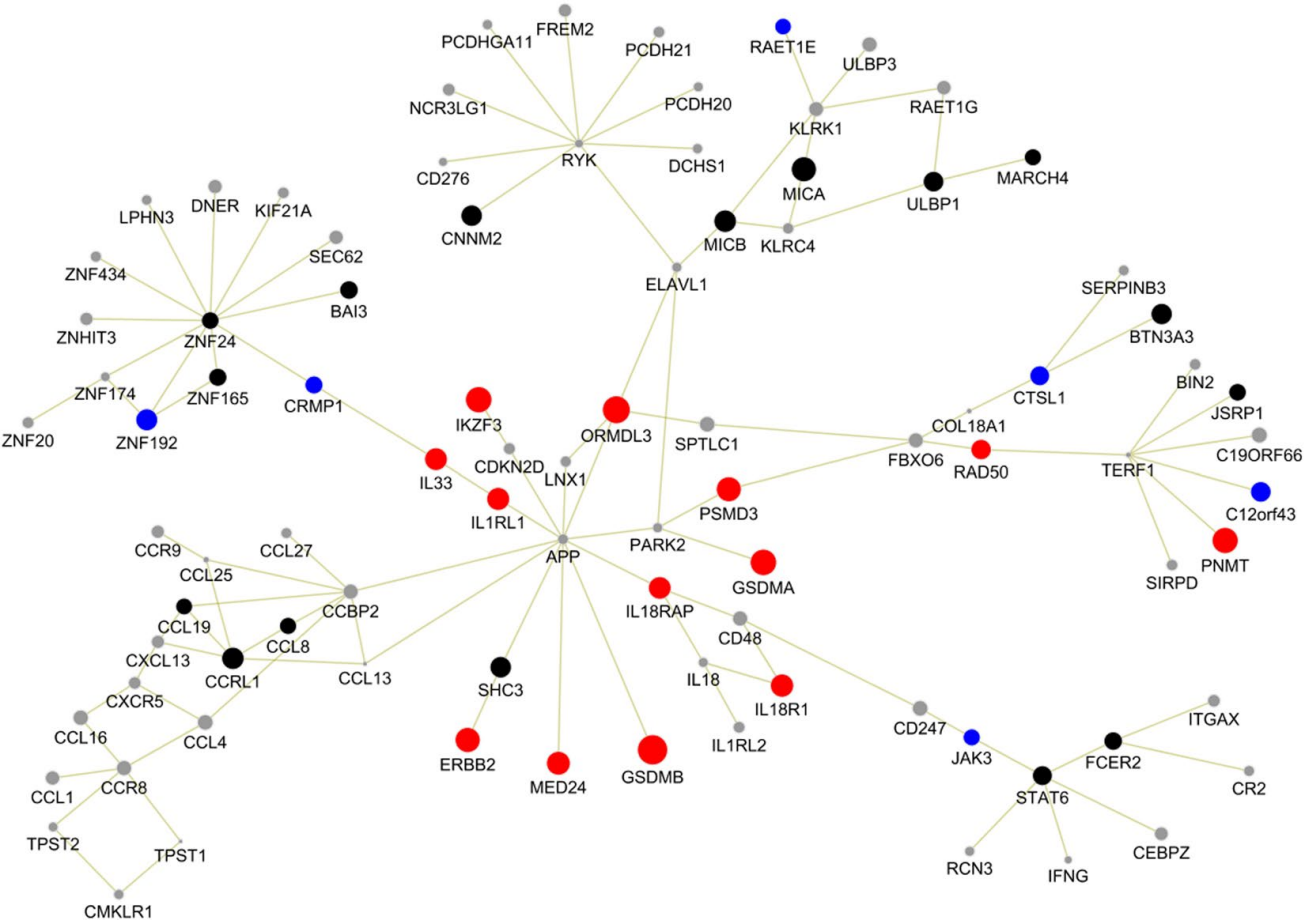
The gene module identified for childhood-onset asthma. The red coloured nodes represent genes at known asthma associated loci and nominally significant in both META1 and META2 datasets; the blue coloured nodes represent new module genes that are nominally significant in both META1 and META2 datasets; the black coloured nodes represent new module genes that are nominally significant in either dataset; the grey coloured nodes are not significant. The node size indicated the strength of the association (the maximum z-score of its corresponding gene in the two datasets).

To identify consistent modules between the two datasets, we computed all pairwise module similarities across datasets. The similarity between two modules was defined by the proportion of genes they share (see Methods). Among a total of 1,072,904 module pairs, 95% had low similarity of less than 0.20 but 77 pairs had similarity over 0.40, showing notable consistency of the involved modules (Supplementary Fig. S3). In order to obtain the most consistent results, we selected the top 10 module pairs with highest pairwise similarities. These module pairs had similarity ranging from 0.42 to 0.63. The modules belonging to these 10 module pairs were further merged within each dataset, resulting in a single connected subnetwork of 171 genes with 243 interactions in META1 and 201 genes with 289 interactions in META2 (Supplementary Fig. S4). Finally, we took the intersection of the two subnetworks and obtained a final module of 91 genes with 106 interactions (Fig. 2 and Supplementary Table S1).

### Module assessment

The identified gene module showed significant association with childhood-onset asthma, with *P*_*assoc*_ < 10^−5^ in both META1 and META2 (using 100,000 circular genomic permutations). This module had a significantly higher score than expected by chance, with *P*_*zm*_ < 10^−5^ and ${P}_{{z}_{m}}^{mhrw} < 7.9\times {10}^{-5}$ evaluated in both META1 and META2, where *P*_*zm*_ was computed by comparing the identified module score with the scores of 100,000 topology-free random modules, and ${P}_{{z}_{m}}^{mhrw}$ with the scores of 12,709 random modules generated by the modified Metropolis-Hasting Random Walk (MHRW) algorithm that takes into account the connection among genes (see Methods for details). The gene module was also enriched in genes that are nominally significant in at least one dataset (${P}_{sig}^{hyper}=2.3\times {10}^{-12}$ and ${P}_{sig}^{mhrw} < 7.9\times {10}^{-5}$). Moreover, the number of pairwise connections among nominally significant genes was significantly higher for genes in the selected module than for genes outside the selected module (*P*_*con*_ = 3.0 × 10^−5^), implying more functional relatedness among the selected genes.

Out of the 91 genes that belonged to the selected gene module, 19 genes had nominally significant *P-*value in both META1 and META2 datasets. These 19 genes included 13 genes at 4 loci found significantly associated with asthma in previous GWAS (2q12, 5q31, 9p24.1, 17q12-q21)^4, 6^, and six genes at six distinct loci that are novel: *CRMP1* (4p16.1), *ZNF192* (6p22.1), *RAET1E* (6q24.3), *CTSL1* (9p21.33), *C12orf43* (12q24.31) and *JAK3* (19p13-p12). Among the other 72 genes, 16 genes were nominally significant in one dataset (META1 or META2) while the remaining genes were connected to the nominally significant genes (Supplementary Table S1). The overall module contained a core subnetwork and five peripheral subnetworks connected to the core. The core subnetwork included genes at the known 2q12, 9p24.1 and 17q12-21 loci that were all connected through the *APP* gene (amyloid beta precursor protein) which occupies a central position in this subnetwork. The five peripheral subnetworks, each harbouring multiple nominally significant genes, were brought together through these core genes (Fig. 2). It is of note that the *APP* gene, which encodes the amyloid beta precursor protein, predisposes to dominant forms of Alzheimer’s disease (AD) but also harbours rare variants with a protective effect on AD^12^. This protein is cleaved by secretases to form a number of peptides, some of which contribute to amyloid plaques in the brains of patients with AD while others have bactericidal and antifungal activities. We noticed that *APP* is a hub gene in the scored-PPI network, as it has the second highest number of interactors (1,727). Nonetheless, two elements show that it was identified in the final module not only for its “hubness”, but also for its interactions with genes strongly associated with COA. First, *APP* was present in 97% of the raw modules generated by DMS, while two other hub genes with comparable number of interactors, *NRF1* (2,174 interactors) and *SUMO2* (1,098 interactors), were included in less than 5% of the raw modules. Second, setting the score of all direct interactors of *APP* in the identified module to zero led to a dramatic decrease from 97% to 4% of the raw modules containing *APP*. This demonstrates that *APP* was identified in the final module mainly for its interactions with strongly COA-associated genes. This link between *APP* and asthma-associated genes suggests potential relationship between AD and asthma that will be further discussed.

### Functional clustering and annotations of the identified module genes

**Figure 3:**
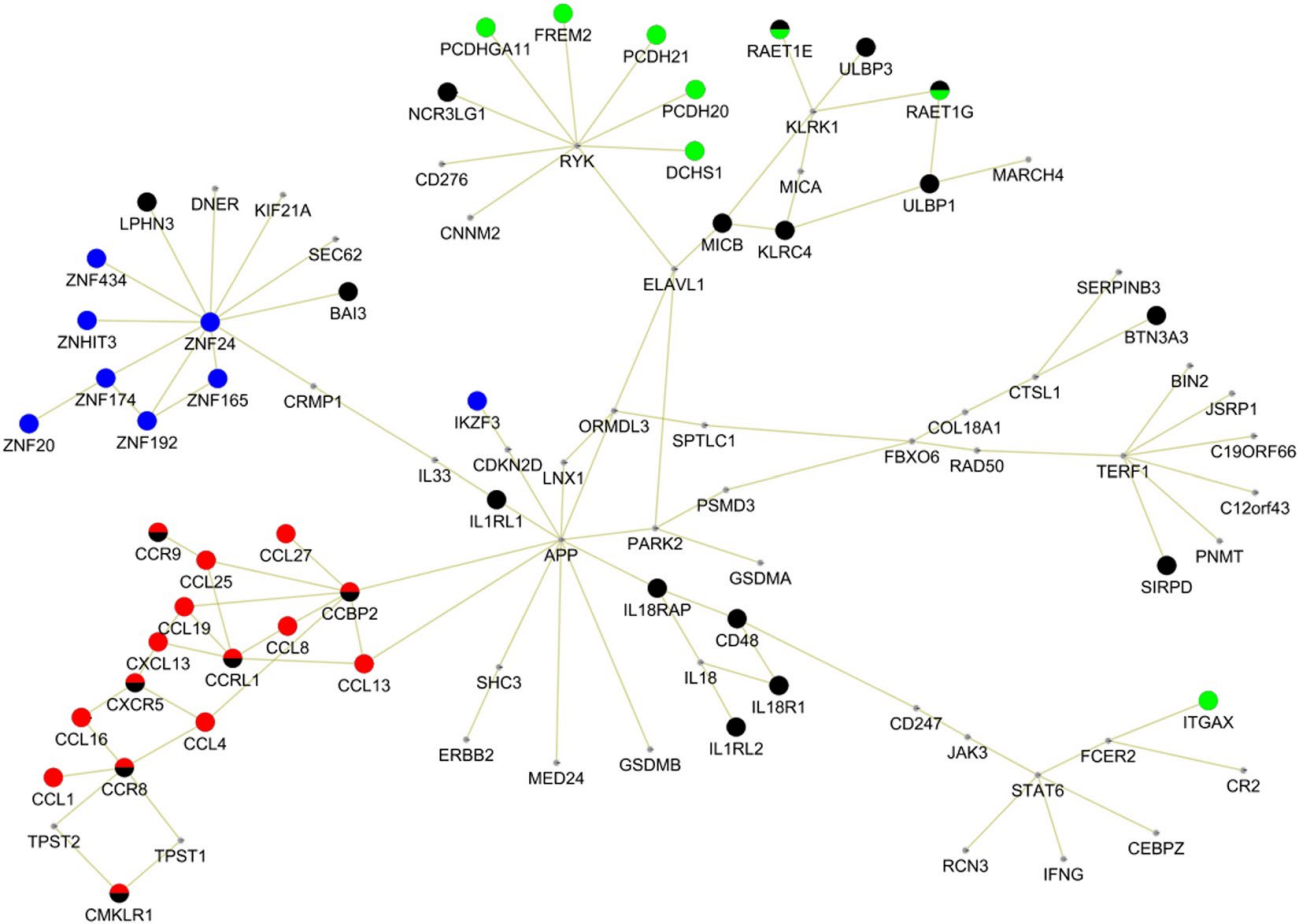
Clusters of functionally-related genes in the COA module. Four genes cluster including a total of 44 out of 91 module genes were identified using DAVID^13, 41^. The genes coloured in black belong to the Immune Response cluster; the genes coloured in red belong to the Chemokines/Chemotaxis cluster; the genes coloured in green belong to the Cadherins/Cell-adhesion cluster and the genes coloured in blue belong to the Zinc finger proteins/Transcription regulation cluster. Genes belonging to multiple clusters are marked by mixed colours.

The functional and biological relatedness of the module genes were explored using the gene functional classification tool of DAVID^13^. This tool clusters genes into functionally related groups according to gene-to-gene annotation similarities using over 75,000 terms from 14 annotation sources (including KEGG, Gene ontology etc.), allowing a much more comprehensive analysis than enrichment analysis based solely on Gene ontology categories or KEGG pathways. We identified four functional gene clusters which altogether included 48% of the module genes (Fig. 3 and Table 1). The largest cluster (cluster 1) encompassed 22 genes scattered across the module while the three other functional clusters showed almost complete overlap with the peripheral subnetworks. These clusters were annotated by the most representative terms that were “immune response”, “chemokines/chemotaxis”, “cadherins/cell-adhesion” and “zinc finger proteins/transcription regulation”, respectively (Table 1).

**Table 1:** Clusters of functionally-related genes characterised in the childhood-onset asthma module using DAVID^13, 41^.

Gene cluster	Functional annotation	Number of genes	List of genes in a cluster
1	Immune response	22	*BAI3, BTN3A3, CCBP2, CCR8, CCR9, CCRL1, CD48, CMKLR1, CXCR5, IL18R1, IL18RAP, IL1RL1, IL1RL2, KLRC4, LPHN3, MICB, NCR3LG1, RAET1E, RAET1G, SIRPD, ULBP1, ULBP3*
2	Chemokines/Chemotaxis	15	*CCBP2, CCL1, CCL13, CCL16, CCL19, CCL25, CCL27, CCL4, CCL8, CCR8, CCR9, CCRL1, CMKLR1, CXCL13, CXCR5*
3	Cadherins/Cell-adhesion	8	*DCHS1, FREM2, ITGAX, PCDH20, PCDH21, PCDHGA11, RAET1E, RAET1G*
4	Zinc finger proteins/Transcription regulation	8	*IKZF3, ZNF165, ZNF174, ZNF192, ZNF20, ZNF24, ZNF434, ZNHIT3*

## Discussion

Network-assisted analysis provides a powerful approach to explore the joint effects of multiple genetic factors on disease and to discover new candidate genes that are missed by single-marker analysis. This is of particular interest for asthma where the number of loci reported by GWAS is relatively small as compared to other common diseases. By integrating the results of two large-scale meta-analyses of childhood-onset asthma with a comprehensive protein-protein interaction network, we identified a gene module of 91 genes that significantly influences COA. This module includes known genes and novel promising candidates. Functional annotation of this module revealed biologically meaningful processes underlying childhood asthma.

The core of the identified module included 11 genes at the three loci that reached genome-wide significance in the meta-analysis of all 18 Gabriel Consortium childhood-onset GWAS, and were also replicated by many other studies^4^, which demonstrates the validity of our strategy. The connection of these genes with *APP* in the core subnetwork is of great interest and is supported by a number of studies indicating that asthma and Alzheimer’s disease (AD) may share common underlying mechanisms. Epidemiological studies have reported an increased risk of AD and dementia in patients with asthma or other allergic diseases^14, 15^. Genetic factors involved in immune-related and inflammatory processes, which are key in asthma, are associated with AD^16^. Epigenetic signatures for both neuronal and immune-response genes were found in a mouse model of AD and in orthologous regions in humans^17^. It has also been recently suggested, in mouse and worm models of AD, that amyloid-β peptide may play a protective role in innate immunity^18^. Finally, in a mouse model of AD, an asthma drug was found to have a potential beneficial impact in AD by decreasing the levels of amyloid-β peptides^19^. The link between *APP* and asthma genes, as highlighted in our module, can open new routes of research for elucidating the functional role and relationships of these genes in asthma, and also potentially, in AD.

The identified module highlighted six novel genes that were nominally significant in both META1 and META2 datasets. The functions of at least four of these genes make them strong candidates for asthma. *RAET1E* (retinoic acid early transcript 1E) belongs to the major histocompatibility complex (MHC) class I-related genes of the *RAETA* family which encode ligands for NKG2D receptor, known to be involved in innate and adaptive immune responses. In the identified module, *RAET1E* is connected to the NKG2D encoding gene *KLRK1*, and through *KLRK1*, to several genes of *MIC* and *RAET/ULBP* families which all encode NKG2D ligands that appear on the surface of stressed cells, such as virus-infected cells^20^. Some of these genes were also nominally significant. This clearly illustrates the usefulness of network analysis in pointing out a set of functionally-related genes that may collectively influence COA, while, individually, they only show nominal association or even no association. Another candidate is *CTSL1*, which encodes a proteinase that acts on the alpha-1 protease inhibitor, a major controlling element of neutrophil elastase activity associated with allergic airway inflammation and severe asthma^21^. *JAK3* (19p13-p12) encodes Janus kinase 3, a member of the Janus kinase family of tyrosine kinases that is predominantly expressed in immune cells and involved in cytokine receptor-mediated intracellular signal transduction. *CRMP1* (collapsin response mediator protein 1) encodes a member of a family of cytosolic phosphoproteins that are expressed in the nervous system but is also an interactor of IL33, a cytokine with a prominent role in asthma^6^. The other two potential candidates, *C12orf43* (chromosome 12 open reading frame 43) and *ZNF192* (encoding a zinc finger protein) have less well known functions.

Our network-assisted analysis is based on the assumption of “guilt-by-association” which states connected genes are usually participating in the same or related cellular functions. We certified the validity of this assumption through gene function clustering analysis. We characterized four gene clusters involving nearly half of the module genes. Three of these clusters, annotated as “chemokines”, “cadherins” and “zinc finger proteins”, are topologically overlapping with three peripheral subnetworks of the module and are related to the core subnetwork in various ways. The “chemokines” cluster is made of chemokines and their receptors, which are all interconnected in the PPI and are involved in several biological processes that may contribute to asthma pathogenesis, such as recruitment and activation of immune and inflammatory cells, collagen deposition and airway wall remodeling^22^. One component of this cluster, CCBP2 (chemokine binding protein 2), shows direct interaction with the core protein APP and two nominally significant chemokines, CCL8 and CCL19. It is also part of the broad functional immune response cluster. Furthermore, CCBP2 was found associated with CCL2 chemokine levels in the cerebrospinal fluid of Alzheimer patients^23^. The “cadherins” cluster includes mainly protocadherins that are part of the cadherin superfamily involved in cell adhesion^24^. While protocadherins may contribute to the defect in epithelial barrier function observed in asthma, as suggested for some of them^25^, a role of other protocadherins in asthma is still unknown. These proteins, which are interacting in the network, are also functionally clustering together with RAET1E, which is a strong asthma candidate (as described above) and is part of the broad immune response cluster. The “zinc finger proteins” cluster includes proteins that show widespread binding to regulatory regions across the genome^26^ but their role in regulating expression of cytokines and other inflammatory proteins, as other transcription factors known to be implicated in asthma^27, 28^, remains to be established. The zinc finger proteins cluster is linked to the core subnetwork in two ways: through direct interaction of CRMP1 protein, encoded by a nominally significant candidate, with IL33, known to be associated with asthma and part of the core network, and through functional relationship with IKZF3, a zinc finger transcription factor regulating B lymphocyte differentiation, encoded by a gene at the known 17q12-q21 asthma locus^5, 6^ and part of the core network. All these results show that the integration of association signals with protein-protein interaction network plus functional clustering analysis bring together interrelated biologically meaningful processes that may underlie the risk for asthma.

The analysis strategy proposed in this study included a novel, exact and efficient algorithm to compute gene-level *P-*values from SNP-level *P-*values. Although other existing methods also allow such computation, including VEGAS2^29^, MAGMA^30^ and PASCAL^31^, these methods use raw genotype data, or an external reference SNP panel (e.g., Hapamap2 or 1000 Genomes panel) when the original genotype data are unavailable, to compute the correlation among SNP statistics. Use of an external reference SNP panel has two limitations. First, some SNPs from a GWAS may not be part of the reference panel, thus will be discarded from the analysis and therefore the results of corresponding genes will be affected. Second, the LD structure estimated from an external reference SNP panel may not always reflect the true correlations among SNP *P*-values, especially in datasets from large genetic consortiums which are usually composed of different populations. Advantageously, fastCGP keeps all SNPs for analysis and utilizes the LD pattern existing in the input data. Though fastCGP is permutation-based in nature, the exact implementation we proposed does not require generating any CGP sample, and provides the best obtainable *P-*value without relying on a limited number of samples as required by typical permutation test procedures. It also avoids variation of the outcomes compared to simulation-based methods, such as VEGAS2. Our analytical implementation of fastCGP reduces considerably the computational time as compared to simulation-based approaches (see Supplementary Information). A potential limitation of fastCGP is that the circular genomic permutation strategy it implements corrects each gene-level *P*-value for the average LD across the genome. Thus, genes with higher LD level than average will be undercorrected while genes with lower LD will be overcorrected. However, this issue of within-gene LD variation may not be so critical for fastCPG as it only uses the best SNP *P*-value instead of all SNP *P*-values to compute the gene *P*-value. Moreover, comparison of fastCGP with VEGAS2 and MAGMA showed strong correlation between the results of fastCGP and the other two methods (use *-bestsnp* sub-model for VEGAS2 and *-snp-wise = top,1* sub-model for MAGMA. See Supplementary Information for details).

Besides correlations between SNPs within a gene, linkage disequilibrium may extend over a broad genomic region and create correlations between gene-based *P*-values of nearby genes. This issue of gene clusters has been addressed in pathway-based analysis^32, 33^, where sensitivity analysis is usually done by including and excluding such genomic regions (e.g., HLA region). For network analysis, such sensitivity analysis is not easy to implement because of the dynamic nature of the module search algorithm and the risk of dismantling the network by removing a few genes. Novel strategies that enable to address this linkage disequilibrium issue deserve further investigation.

It is well known that analyses performed at a genome-wide scale are prone to high rates of false positives. To ensure the reliability of findings, strict criteria have been established for reporting GWAS results, such as the use of a stringent threshold to declare significance and replication of results. However, such criteria are less well defined in pathway and network analyses. It is also worth noting that most module searching methods, including DMS, are based on heuristic or greedy algorithms which do not guarantee finding the module with highest score but may include irrelevant genes by chance^34^. To reduce the amount of false positive findings caused by either the noise from input data or by the module search method, previous network-based studies have used two or more GWAS datasets and implemented cross-evaluation strategies to identify modules showing consistent association signals across datasets^35, 36^. In the current study, we used two large GWAS datasets, resulting from a meta-analysis of nine GWAS each, and designed a parallel dense-module search and consistency-based cross-selection strategy to increase the reliability of results. The consistency, defined in terms of similar gene composition between modules obtained from two independent datasets, has the ability to take into account the module topology, and hence to select modules that share genes with association signals and genes closely connected to these disease-associated genes, both of which may play a role in COA susceptibility. We also compared our parallel strategy to a non-parallel strategy by repeating network analysis using a single GWAS dataset made of the meta-analysis results of all 18 childhood-onset asthma GWAS. We selected the same number of genes (91) using the approach proposed by dmGWAS^11^. We found the non-parallel strategy selected less genes that were replicated across datasets (it selected 13 genes nominally significant in both META1 and META2 while the parallel strategy selected 19 such genes), and were less functionally related based on DAVID analysis (the non-parallel module contains two gene clusters that includes 9% of the module genes while the parallel module contains four clusters that includes 48% module genes). This indicates the advantage of using a parallel strategy at least for these asthma data but further studies applied to various datasets are needed to confirm these findings.

As for many other module selection strategies^11, 36^, our strategy involves the choice of a cut-off defined as the number of consistent modules across datasets to be selected for downstream analysis. We chose the 10 module pairs (out of 1,072,904 pairs) having highest pairwise module similarity between the two datasets. Although selection of more module pairs may allow including additional candidate genes, it may also increase the complexity of downstream analysis. When we repeated the analysis by loosening the cut-off of module pairs selection, i.e. by selecting 15 or 20 module pairs instead of 10 pairs, no additional relevant information was obtained. Indeed, only one gene out of a maximum of 34 additional genes selected in the final module was nominally significant in the two datasets and belonged to a well-known asthma-associated region on chromosome 17q12-q21. In addition, clustering analysis using DAVID did not identify any additional functional cluster. This shows that our choice of the 10 most consistent module pairs is reasonable. Nonetheless, modules that contribute to asthma susceptibility but did not rank in the top modules may have been missed. More sophisticated methods that allow finding an optimal similarity cut-off deserve further consideration.

In summary, we have derived a comprehensive network-assisted analysis strategy and identified a module of 91 genes significantly contributing to asthma risk. As part of this strategy, we developed an exact and efficient gene-based method (fastCGP) to compute gene-level *P-*values, and a parallel dense-module search and cross-selection strategy to identify an asthma-associated module, both of which are key elements of our network analysis. This study was able to confirm known asthma genes and to pinpoint novel relevant candidates. It also highlighted many links between subnetworks of the identified module and functional relationships both within and across subnetworks, thus providing new clues for future research in both the genetics and pathogenesis of asthma.

## Methods

### Childhood-onset asthma (COA) GWAS datasets

We used two COA GWAS datasets that consisted of the outcomes of meta-analysis of nine COA GWAS each. These GWAS were part of the European GABRIEL Consortium and have been described in detail elsewhere^6^. Briefly, in these studies, asthma was considered to be present if it had been diagnosed by a physician and childhood-onset asthma was defined as the presence of the disease in a person younger than 16 years. The genotyping of all datasets was performed using the Illumina Human610-Quad Beadchip. Imputations were done using MACH 1.0 and Hapmap2 reference panel (release 21). After quality control filtering (imputation quality score ≥0.50 and minor allele frequency ≥1%), a total of 2,370,689 autosomal SNPs were kept in the analysis. A total of 18 GABRIEL childhood-onset asthma GWAS, all of European-ancestry, were randomly split into two datasets of 9 GWAS each of similar size (3,031 cases/2,893 controls in the first dataset and 2,679 cases/3,364 controls in the second dataset). A random-effect meta-analysis was performed in each dataset using Stata™ V10.0 (distributed by Stata Corporation, College Station, Texas, USA). The outcomes of these meta-analyses (single-SNP *P-*values) were named META1 and META2 respectively.

### Computing gene-level *P-*values by fastCGP

To perform network analysis, gene-level *P-*values representing the significance of each gene for association with COA were computed. We developed an exact and efficient algorithm, named fastCGP, to calculate gene-level *P-*values from SNP-level *P-*values. To start, SNPs were mapped to genes (between the start site and 3′-untranslated region of each gene) using dbSNP Build 132 and human Genome Build 37.1. Each gene-level *P-*value *P*_*g*_ is taken as the best SNP *P-*value among all SNPs mapped to the gene. These *P-*values are biased by gene length (number of SNPs being mapped) since genes with more SNPs mapped tend to have a lower best SNP *P-*value by chance. We corrected for such bias using permutation-based approach. To keep similar patterns of LD among SNPs in the permutated data as in the original data, we implemented the Circular Genomic Permutation (CGP) strategy^37^. Briefly, CGP considers the genome as a circle, starting from chromosome 1 and ending at chromosome 22. SNP-level *P-*values of a GWAS are ordered on the circle according to the position of the SNPs. A CGP sample can be generated by rotating the *P-*values from a randomly chosen position and reassigning these *P*-values to each SNP. As in a typical permutation test, we defined the corrected gene-level *P-*value as *P*_corrected_ = 1 − *l*/(*L* + 1), where *L* is the total number of CGP samples, and *l* is the number of samples with *P*_*π,g*_ > *P*_*g*_ (*P*_*π,g*_ is the best SNP *P*-value of gene *g* based on a permutation sample). Particularly, in contrast to general permutation tests classically relying on a limited number of samples, we considered all non-repeating CGP samples in order to obtain the best obtainable *P-*value within this permutation-based framework. In such a case, *L* becomes the total amount of SNPs placed on the circle (hence the number of SNPs in a GWAS), while *l* can be calculated analytically and efficiently without generating any CGP sample. The detail of this analytical approach along with an illustrative example is given in Supplementary Information. We implemented fastCGP in R and made it publically available at https://github.com/YuanlongLiu/fastCGP.

We applied fastCGP separately to META1 and META2 to compute gene-level *P-*values. A total of 24,120 genes were analysed for each dataset.

### Overloading gene-level signals to protein-protein interaction network

We converted gene-level *P-*values to z-scores by *z*_*i*_ = Φ^−1^(1 − *p*_*i*_), where Φ is the cumulative distribution function of the standard normal distribution. We downloaded the human protein-protein interaction network (PPI) from the Protein Interaction Network Analysis platform^38^ (release of May 21, 2014). It integrates annotated protein-protein interaction data from six public curated databases (IntAct, BioGRID, MINT, DIP, HPRD and MIPS/MPact). To reduce the uncertainty of network data, we kept only the interactions having experimental evidence. We overloaded gene scores to the PPI to build a scored-PPI for each of META1 and META2.

### Identification of a module enriched with childhood-onset asthma-associated genes

We applied the dense module search (DMS) algorithm implemented in dmGWAS R package^11^ within each scored-PPI to search modules that consist of high score genes. Briefly, DMS defines the score of a module of *k* genes as ${Z}_{m}=\sum \,{z}_{i}/\sqrt{k}$. It grows a module from a seed gene and adds the neighbouring gene that can lead to the maximum increment of the module score. Module growth terminates if adding neighbouring genes does not yield an increment of module score by at least *Z*_*m*_ × 0.1.

Due to the nature of DMS algorithm that uses every gene in the scored-PPI as a seed to grow module, thousands of modules might be generated and there is extensive overlap among them. To reduce such redundancy, we hierarchically merged the raw modules within each dataset until all pairwise Dice similarity were less than 0.50, where the Dice similarity between two modules *A* and *B* is defined as $s(A,B)=2|A\cap B|/(|A|+|B|)$ ^39^. Here |•| represents the number of genes in a module; $A\cap B$ represents the genes shared by module *A* and module *B*.

The original dmGWAS paper suggested selecting 1% of the modules with highest normalized module score. In our study, we were rather interested in modules generated independently from separate datasets but having similar compositions across datasets, thus to improve the reliability of results. Module consistency was assessed by computing all pairwise module similarities, with one module from META1 and another module from META2. We selected the 10 module pairs with highest pairwise similarities and the selected modules were then merged within each dataset. The final module was constructed by taking the shared genes between the two merged modules.

### Module assessment

We performed various types of statistical tests to assess different features of the final gene module. First, we assessed whether the module is significantly associated with COA, using META1 and META2 respectively. The null distribution of the module score was estimated by permuting the SNP-level *P-*values *n* = 100,000 times through CGP that takes into account the genomic structure. For each CGP permutation, gene-level *P-*values were recalculated using fastCGP and module scores were computed. The *P-*value of association of the module with COA was defined as ${P}_{assoc}=(\#\{{Z}_{m(s)}\ge {Z}_{m}\}+1)/(n+1)$.

Second, we evaluated whether the module selected by our strategy has a higher score than by chance. Two sets of random modules were generated as background. The first consists of *n* = 100,000 modules sampled from the scored-PPI without considering their connections (topology-free). Each module has the same number of genes as that of the module under test. The corresponding *P-*value is ${P}_{{z}_{m}}=(\#\{{Z}_{m(s)}\ge {Z}_{m}\}+1)/(n+1)$. The second set of random modules was generated by taking the connection among genes into account. Specifically, we constrained the genes to be connected with each other (directly or indirectly) within each random module, so that they are more biologically related and is more comparable to the module under test. We inherited the Metropolis-Hasting Random Walk (MHRW) algorithm^40^ to generate random modules (see Supplementary Information). A total of *N* = 12,709 modules were generated. The *P-*value was defined as ${P}_{{z}_{m}}^{mhrw}=(\#\{{Z}_{m(s)}\ge {Z}_{m}\}+1)/(N+1)$. We computed *P*_*zm*_ and ${P}_{{z}_{m}}^{mhrw}$ in META1 and META2 respectively.

Third, we assessed whether the selected module is enriched in genes nominally significant in at least one dataset. These genes have high probability of association with asthma hence are of high interest. We used a hypergeometric test to assess whether the selected module contains a higher proportion of such genes than the background. The *P-*value is defined by ${P}_{sig}^{hyper}=1-{F}_{h}(k;K,n,N)$, which is the tail probability of a hypergeometric distribution that a module of *K* genes contains at least *k* nominally significant genes, while the whole scored-PPI of *N* genes contains *n* nominally significant genes. We also evaluated the significance by comparing with the MHRW random modules. The *P-*value was defined as ${P}_{sig}^{mhrw}=(\#\{k(s)\ge k\}+1)/(N+1)$, where *k*(*s*) is the number of nominally significant genes in a random module.

Finally, we evaluated whether the nominally significant genes in the selected module are more interconnected than those outside the module. We sampled *n* = 100,000 times the same number of genes from the unselected nominally significant genes and computed the amount of the connections between them. The *P-*value is ${P}_{con}=(\#\{e(s)\ge e\}+1)/(n+1)$, where *e* is the number of connections between nominally significant genes in the identified module, while *e*(*s*) is the corresponding number in a sample.

### Functional clustering and annotation identified genes

To explore the functional relatedness of genes belonging to the selected module, we conducted the gene functional classification analysis provided by DAVID Bioinformatics Resource^41^. This tool generates a gene-to-gene similarity matrix based on shared functional annotation profiles using over 75,000 terms from 14 annotation sources of different types (ontology, protein domain/family, pathways, functional categories, or disease association) and use a heuristic fuzzy multiple-linkage partitioning to identify functionally related gene clusters. The gene functional classification analysis was run for the list of genes in the final module. We set the genes mapped to the PPI as background and used the default parameters of DAVID in our analysis.

## Electronic supplementary material

Supplementary Info
