# Supplementary material for: Network-assisted analysis of GWAS data identifies a functionally-relevant gene module for childhood-onset asthma
Source: Sci Rep. 2017 Apr 20;7:938. doi: 10.1038/s41598-017-01058-y (PMC5430538; doi:10.1038/s41598-017-01058-y)
Supplement: Supplementary file 1 — Supplementary Info [file 41598_2017_1058_MOESM1_ESM.pdf]

SUPPLEMENTARY INFORMATION

Network-assisted analysis of GWAS data identifies a functionally-relevant gene module for childhood-onset asthma

Y. Liu[1,2*+], M. Brossard[1,2+], C. Sarnowski[1,2], A. Vaysse[1,2], M. Moffatt[3], P. Margaritte-Jeannin[1,2], F. Llinares-López[4], M.H. Dizier[1,2], M. Lathrop[5], W. Cookson[3], E. Bouzigon[1,2], F. Demenais[1,2*]

[+]These two authors contributed equally to the work

[1]INSERM, Genetic Variation and Human Diseases Unit, UMR-946, Paris, France

[2]Université Paris Diderot, Université Sorbonne Paris Cité, Institut Universitaire d'Hématologie, Paris, France

[3]Genomic Medicine Section, National Heart Lung Institute, Imperial College London, London, UK

[4]Machine Learning and Computational Biology Lab, Department of Biosystems Science and Engineering, ETH Zürich, Basel, Switzerland

[5]McGill University and Genome Québec Innovation Centre, Montréal, Québec, Canada.

Correspondence should be addressed to Y. Liu (email: yuanlong.liu@inserm.fr) or F. Demenais (email: florence.demenais@inserm.fr)

Table of content

Supplementary Figure S1. Double Manhattan plot of gene-level *P*-values in META1 and META2.... 1

Supplementary Figure S2. Distribution of pairwise module similarities before and after merging the raw modules generated by the Dense Module Search algorithm. 2

Supplementary Figure S3. Distribution of pairwise module similarities between modules in META1 and modules in META2. 3

Supplementary Figure S4. Consistent gene modules between META1 and META2. 4

Supplementary Figure S5. An illustrative example of fastCGP. 5

Supplementary Figure S6. Comparison of gene-level *P*-values obtained by fastCGP and VEGAS2 ... 6

Supplementary Figure S7. Comparison of gene-level *P*-values obtained by fastCGP and MAGMA .. 7

Supplementary Table S1. Gene-level *P*-values in META1 and META2 datasets for the 91 genes in the final childhood-onset asthma module 8

Supplementary Methods 1: computing gene-level *P*-values via fastCGP. 11

Supplementary Methods 2: generating random modules via Metropolis-Hasting Random Walk algorithm. 13

References... 13

Supplementary Figure S1. Double Manhattan plot of gene-level *P*-values in META1 and META2. Gene-level *P*-values were computed from SNP-level *P*-values using fastCGP. META1 and META2 correspond to the results of meta-analysis of 9 COA GWAS each. The GWAS are part of the GABRIEL asthma consortium (ALSPAC, BAMSE, ECRHS, MAS/MAGICS, SLSJ, TOMSK, UFA, CAPPS studies for META1; B58C, BUSSELTON, EGEA, GABRIEL Advanced Surveys, KSMU, MRCA-UKC, PIAMA, SAPALDIA, SAGE studies for META2; see Moffatt et al[1] for details on these studies)

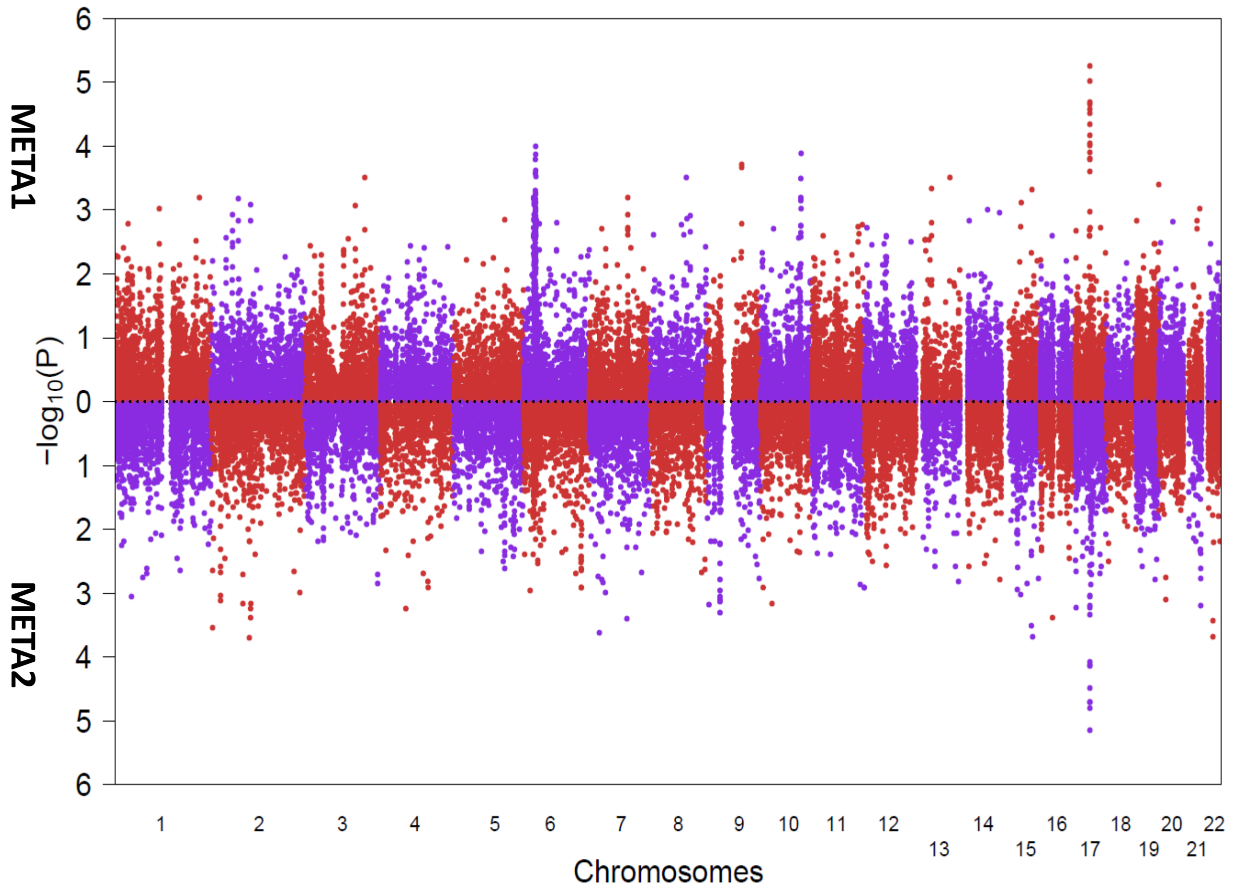

Supplementary Figure S2. Distribution of pairwise module similarities before and after merging the raw modules generated by the Dense Module Search algorithm. The pairwise module similarities (indicating overlaps between modules) were remarkably reduced in both META1 (left panel) and META2 (right panel) after hierarchically merging similar raw modules

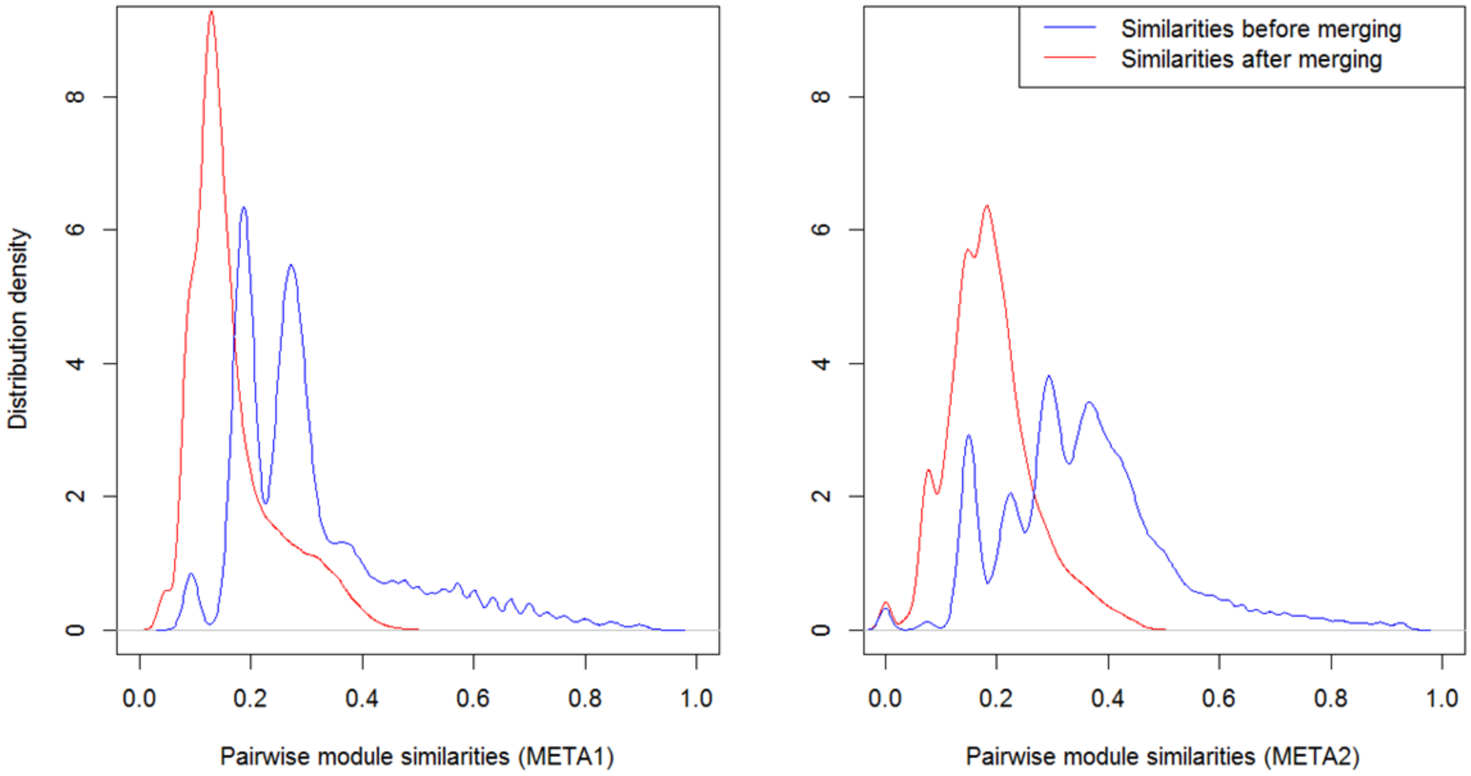

Supplementary Figure S3. Distribution of pairwise module similarities between modules in META1 and modules in META2. A total of 1,072,904 module pairs were constructed. The bins represent histograms of pairwise module similarities. The ticks represent rug plot of the similarities. Each tick represents one pairwise module similarity. The red ticks highlight the 10 highest pairwise module similarities

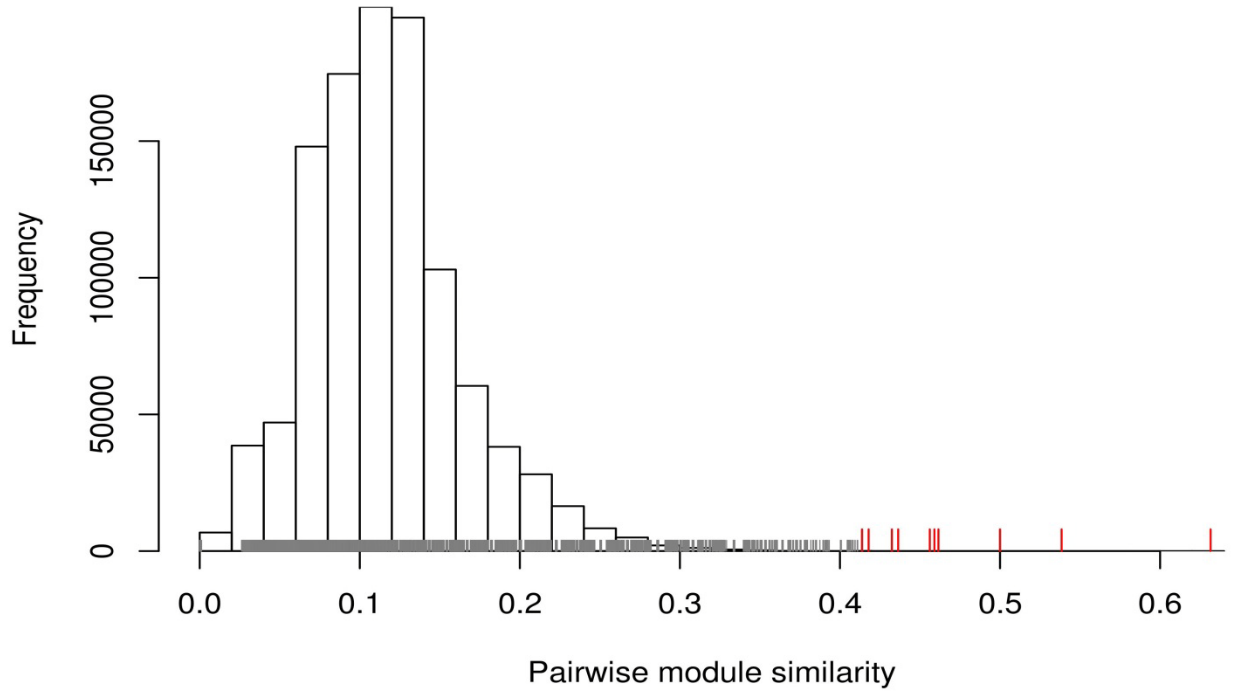

Supplementary Figure S4. Consistent gene modules between META1 and META2. We selected top 10 module pairs showing highest pairwise module similarities among all module pairs between META1 and META2. The involved modules were merged within each dataset, resulting in a subnetwork of 171 genes in META1 (a) and a subnetwork of 201 genes in META2 (b). The intersection of the two subnetworks was retrieved to construct the final module, resulting in a module of 91 genes (nodes in red). The node sizes in this plot are proportional to the gene z-scores

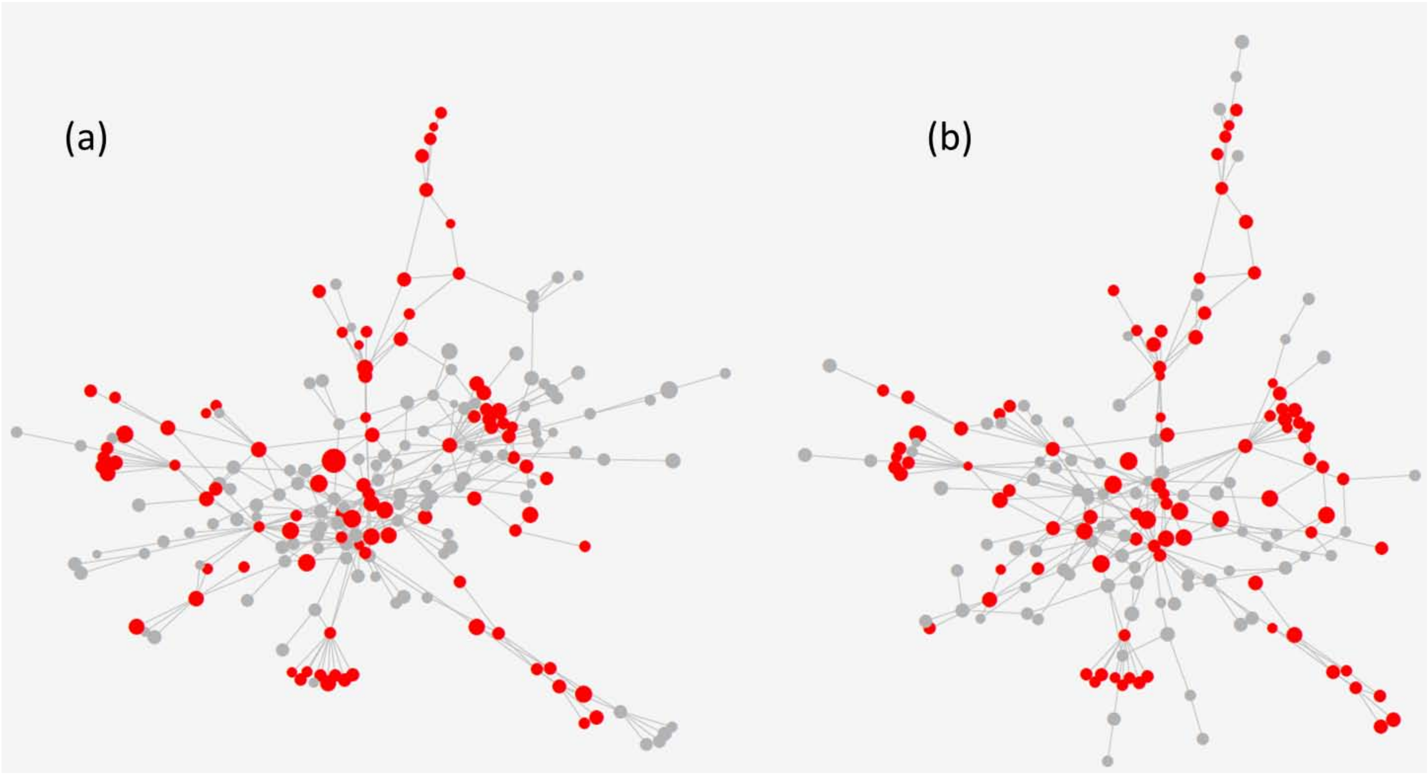

Supplementary Figure S5. An illustrative example of fastCGP. An artificial GWAS result consisting of L=100 SNP *P*-values were created. All *P*-values were set as 0.1 except $P_{11} = 0.05, P_{12} = 0.02, P_{14} = 0.04$, and $P_{18} = 0.07$. These *P*-values are ordered on a circle according to the chromosomal position of corresponding SNPs. A gene g has three SNPs mapped to its genomic region. Its uncorrected *P*-value P_g is set as the minimum SNP *P*-value among the three mapped SNPs ($P_g = P_{18} = 0.07$). There are four extreme SNP *P*-values on the circle P_{11}, P_{12}, P_{14} and P_{18} ($\leq P_g$). The consecutive extreme *P*-value pairs are $P_{11} \sim P_{12}, P_{12} \sim P_{14}, P_{14} \sim P_{18}, P_{18} \sim P_{11}$

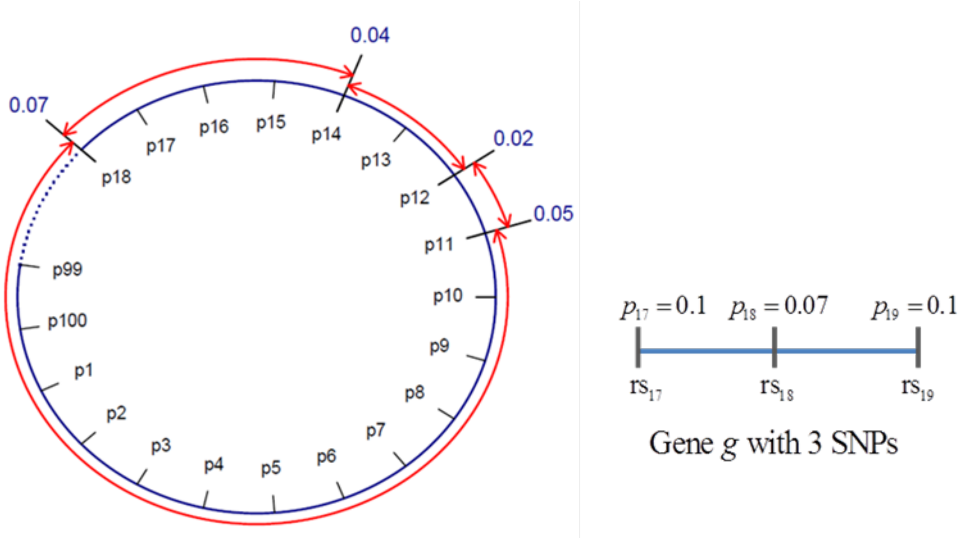

Supplementary Figure S6. Comparison of gene-level *P*-values obtained by fastCGP and VEGAS2[2]. Gene-level *P*-values were computed from asthma META2 dataset using fastCGP and VEGAS2 (-*bestsnp* sub-model). Their results (-log10 (*P*-value)) were compared for each chromosome from chromosome 1 (Chr1) to chromosome 22 (Chr22). The red diagonal lines indicate perfect match (identical) of the two results. ρ represents the Pearson correlation coefficient between the two results

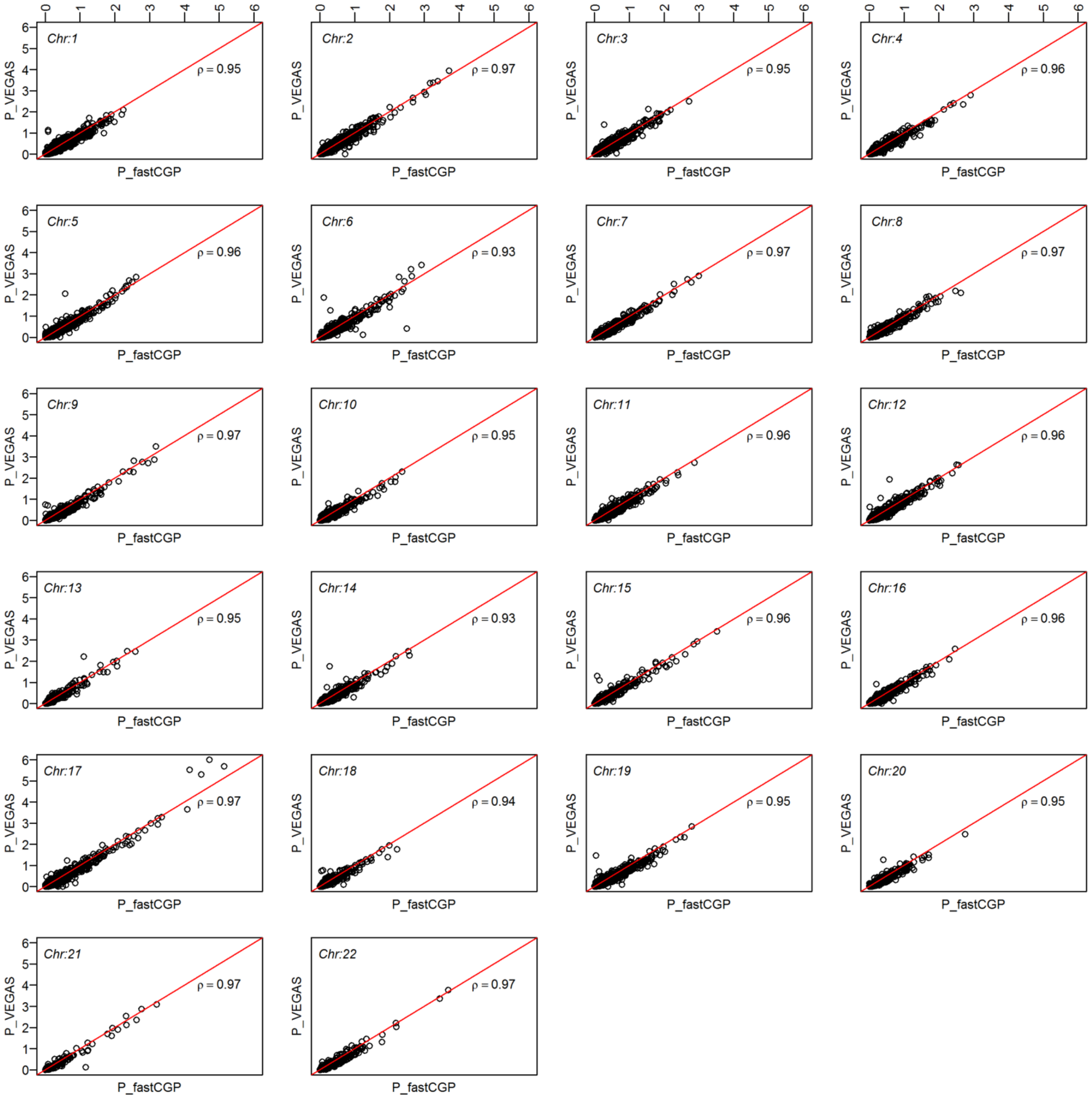

Supplementary Figure S7. Comparison of gene-level *P*-values obtained by fastCGP and MAGMA[3]. Gene-level *P*-values were computed from asthma META2 dataset using fastCGP and MAGMA (*-snp-wise=top,1* sub-model). Their results (-log10 (*P*-value)) were compared for each chromosome from chromosome 1 (Chr1) to chromosome 22 (Chr22). The red diagonal lines indicate perfect match (identical) of the two results. ρ represents the Pearson correlation coefficient between the two results

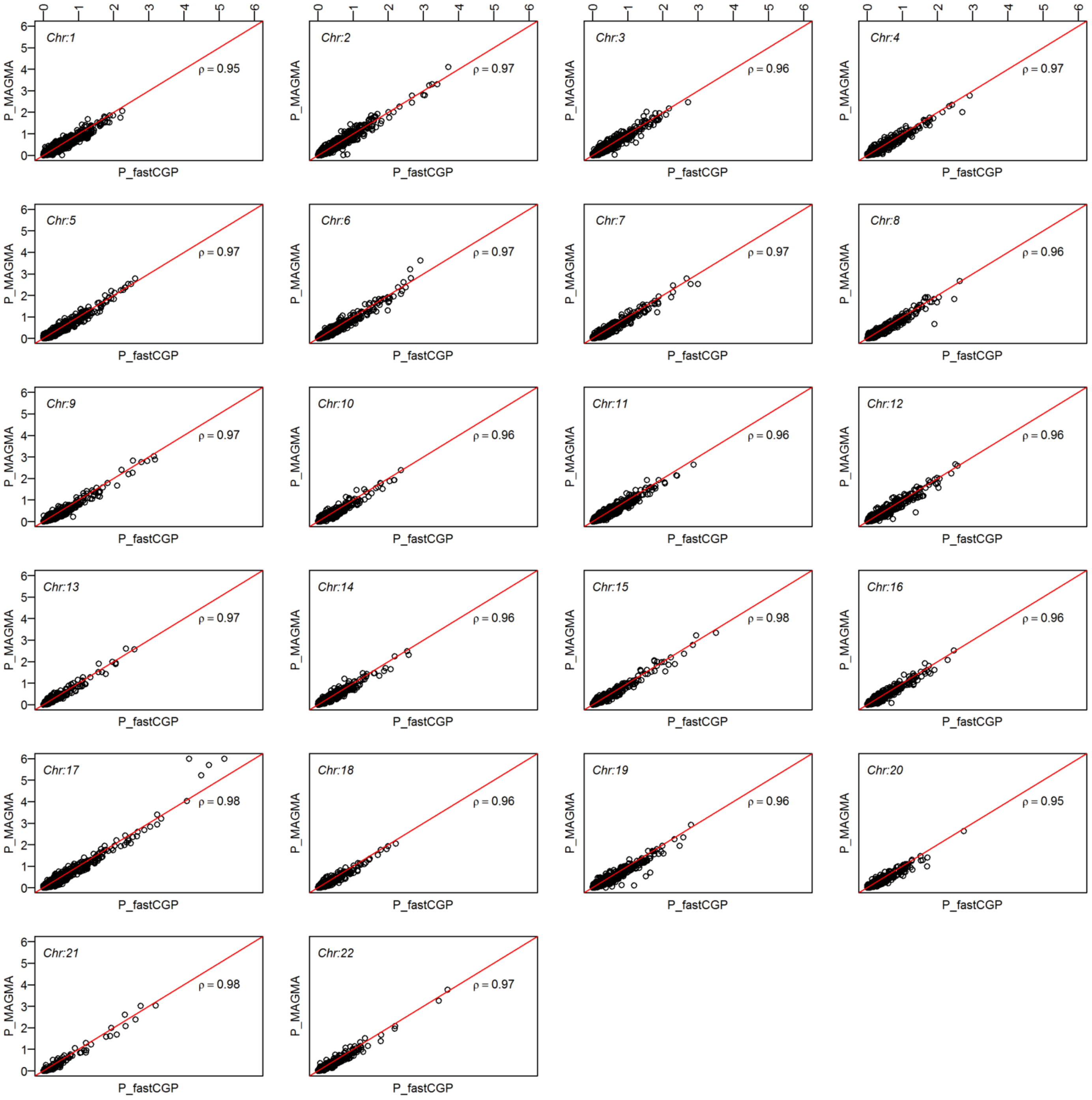

Supplementary Table S1. Gene-level *P*-values in META1 and META2 datasets for the 91 genes in the final childhood-onset asthma module

Chr	Gene	Start	End	# SNPs	META1			META2		
					Best SNP	Best SNP *P*-value	Corrected Best SNP *P*-value (by fastCGP)	Best SNP	Best SNP *P*-value	Corrected Best SNP *P*-value (by fastCGP)
**Genes nominally significant in both META1 and META2**										
2	***IL1RL1***	102 927 962	102 968 497	80	rs4988957	2.9E-05	1.5E-03	rs10192157	2.2E-05	5.6E-04
2	***IL18R1***	102 979 097	103 015 218	48	rs3771166	1.9E-05	8.2E-04	rs1974675	2.4E-05	4.1E-04
2	***IL18RAP***	103 035 254	103 069 025	71	rs6543135	2.6E-03	4.5E-02	rs2310300	2.6E-05	6.9E-04
4	*CRMP1*	5 822 491	5 894 785	60	rs13144677	2.7E-03	4.1E-02	rs10011385	1.3E-03	1.7E-02
5	***RAD50***	131 892 630	131 979 599	35	rs2240032	1.2E-03	1.5E-02	rs2897443	3.8E-04	3.9E-03
6	*ZNF192*	28 109 716	28 125 236	21	rs13205911	6.4E-05	1.2E-03	rs2622321	7.4E-03	4.1E-02
6	*RAET1E*	150 209 601	150 212 097	10	rs9371533	1.1E-02	4.5E-02	rs9371533	1.0E-02	3.6E-02
9	***IL33***	6 241 678	6 257 982	20	rs7019575	4.8E-03	3.2E-02	rs7019575	8.7E-05	6.7E-04
9	*CTSL1*	90 340 974	90 346 384	4	rs2378757	1.8E-03	5.6E-03	rs2378757	6.9E-03	1.4E-02
12	*C12orf43*	121 440 848	121 454 300	10	rs3751150	4.4E-04	3.1E-03	rs3751151	1.2E-02	4.3E-02
17	***PNMT***	37 824 507	37 826 728	1	rs876493	1.3E-07	3.1E-05	rs876493	4.0E-05	7.3E-05
17	***ERBB2***	37 844 393	37 884 915	7	rs1058808	3.5E-06	1.6E-04	rs1058808	1.6E-05	8.2E-05
17	***IKZF3***	37 921 198	38 020 441	48	rs907091	2.5E-15	4.6E-05	rs9909593	1.6E-07	7.1E-05
17	***GSDMB***	38 060 848	38 074 903	12	rs9303281	2.6E-16	5.5E-06	rs2305480	7.5E-08	1.9E-05
17	***ORMDL3***	38 077 296	38 083 854	4	rs12603332	4.6E-15	9.7E-06	rs8076131	7.3E-08	7.2E-06
17	***GSDMA***	38 119 226	38 134 019	6	rs7212938	2.4E-13	2.7E-05	rs3902025	3.2E-07	3.3E-05
17	***PSMD3***	38 137 060	38 154 212	21	rs11655264	1.0E-07	9.6E-05	rs12453334	6.9E-05	5.8E-04
17	***MED24***	38 175 350	38 210 889	21	rs12309	3.0E-06	2.5E-04	rs12451897	1.2E-04	9.2E-04
19	*JAK3*	17 935 591	17 958 841	12	rs2110586	5.8E-03	2.8E-02	rs3212701	1.2E-02	4.5E-02
**Genes nominally significant in either META1 or META2**										
2	*MARCH4*	217 122 585	217 236 750	113	rs1477235	3.0E-02	4.0E-01	rs1510836	1.3E-03	2.8E-02
3	*CCRL1*	132 316 094	132 321 382	2	rs7626622	3.0E-04	8.5E-04	rs7626622	1.7E-01	2.2E-01
6	*BTN3A3*	26 440 763	26 453 643	15	rs13220495	1.8E-04	1.9E-03	rs3846845	1.3E-01	4.3E-01
6	*ZNF165*	28 046 572	28 057 341	9	rs1321505	3.7E-03	1.6E-02	rs203878	2.8E-02	8.8E-02
6	*MICA*	31 371 371	31 383 090	41	rs2844518	3.1E-08	1.0E-04	rs12213831	6.4E-02	4.0E-01
6	*MICB*	31 465 855	31 478 901	26	rs3130614	2.9E-05	7.6E-04	rs2855814	3.5E-01	9.0E-01
6	*BAI3*	69 345 632	70 099 403	770	rs3757043	4.6E-05	1.3E-02	rs17502590	3.7E-02	9.5E-01
6	*ULBP1*	150 285 143	150 294 846	6	rs9478311	1.2E-01	2.8E-01	rs9478311	1.2E-03	3.2E-03
9	*CCL19*	34 689 567	34 691 274	2	rs3176813	4.1E-02	5.5E-02	rs3136658	2.6E-02	3.3E-02
9	*SHC3*	91 628 046	91 793 682	134	rs1331180	2.0E-05	1.6E-03	rs2316280	3.6E-02	4.9E-01

10	*CNNM2*	104 678 114	104 838 241	129	rs943036	2.3E-05	1.8E-03	rs2296569	6.5E-02	6.7E-01
12	*STAT6*	57 489 191	57 505 161	10	rs324015	8.7E-04	5.3E-03	rs1059513	2.0E-02	6.8E-02
17	*CCL8*	32 646 066	32 648 421	2	rs3138036	9.2E-01	9.7E-01	rs3138036	2.2E-02	2.8E-02
18	*ZNF24*	32 912 178	32 924 426	12	rs7239712	4.2E-03	2.2E-02	rs1064753	1.9E-02	7.2E-02
19	*JSRP1*	2 252 252	2 255 344	3	rs7250822	1.1E-02	2.2E-02	rs7250822	2.0E-01	3.2E-01
19	*FCER2*	7 753 643	7 767 032	22	rs2287866	1.4E-03	1.3E-02	rs2303112	1.4E-02	7.8E-02
**Other genes**										
1	*FBXO6*	11 724 150	11 734 411	2	rs747863	6.1E-01	7.5E-01	rs747863	6.1E-02	7.9E-02
1	*CD48*	160 648 536	160 681 585	14	rs3796502	1.6E-02	7.2E-02	rs10489636	8.2E-02	2.9E-01
1	*CD247*	167 399 877	167 487 847	116	rs864537	2.7E-03	6.7E-02	rs864537	3.0E-03	6.0E-02
1	*CR2*	207 627 670	207 663 240	41	rs17258982	4.6E-02	3.2E-01	rs7543913	9.1E-02	5.1E-01
2	*CEBPZ*	37 428 772	37 458 740	12	rs2239650	3.4E-02	1.3E-01	rs11689186	1.0E-01	3.3E-01
2	*IL1RL2*	102 803 433	102 855 811	134	rs17026782	6.6E-02	6.9E-01	rs12987222	1.4E-02	2.4E-01
2	*DNER*	230 222 345	230 579 286	353	rs10192168	1.6E-03	1.1E-01	rs6726280	3.2E-03	1.6E-01
3	*CCR8*	39 371 197	39 375 171	2	rs2853699	5.4E-02	7.2E-02	rs4676633	2.3E-01	3.0E-01
3	*CCBP2*	42 850 964	42 908 775	36	rs13093968	8.3E-03	7.4E-02	rs4396867	4.4E-01	9.7E-01
3	*CCR9*	45 928 019	45 944 667	12	rs17714101	4.5E-02	1.6E-01	rs6441931	2.6E-01	6.7E-01
3	*RYK*	133 875 978	133 969 586	52	rs4280635	8.4E-02	5.4E-01	rs10935104	7.5E-02	4.9E-01
3	*SEC62*	169 684 580	169 716 161	23	rs9813592	1.8E-02	1.1E-01	rs16854694	3.2E-01	8.5E-01
4	*LNX1*	54 326 437	54 457 724	156	rs2117600	2.6E-02	4.4E-01	rs9312642	1.5E-02	2.9E-01
4	*LPHN3*	62 362 839	62 938 168	336	rs17082520	1.3E-02	4.6E-01	rs1497906	8.9E-03	3.4E-01
4	*CXCL13*	78 432 907	78 532 988	27	rs17406477	2.1E-01	7.3E-01	rs355687	2.8E-02	1.6E-01
5	*PCDHGA11*	140 800 537	140 892 546	57	rs11958830	4.5E-02	3.7E-01	rs1423149	1.8E-01	8.1E-01
6	*RAET1G*	150 238 014	150 244 214	4	rs6927913	2.3E-01	4.2E-01	rs9397070	5.0E-02	9.7E-02
6	*ULBP3*	150 385 743	150 390 202	7	rs12202737	3.0E-01	6.3E-01	rs2010212	2.7E-02	7.3E-02
6	*PARK2*	161 768 590	163 148 834	1776	rs4623220	1.6E-03	3.9E-01	rs11966738	1.9E-03	4.0E-01
7	*TPST1*	65 670 259	65 825 438	68	rs778732	3.8E-01	9.8E-01	rs4149463	1.9E-01	8.6E-01
8	*TERF1*	73 921 097	73 959 987	26	rs12334686	2.4E-01	7.7E-01	rs10107605	5.8E-01	9.9E-01
9	*CCL27*	34 661 893	34 662 689	1	rs11575584	6.3E-01	6.1E-01	rs11575584	3.4E-01	3.1E-01
9	*SPTLC1*	94 793 427	94 877 690	74	rs16908106	9.5E-02	6.6E-01	rs12235495	4.1E-03	5.8E-02
10	*PCDH21*	85 954 517	85 977 122	34	rs12781048	2.1E-01	7.6E-01	rs12781048	4.1E-02	2.5E-01
11	*DCHS1*	6 642 558	6 677 080	28	rs11607376	7.1E-02	3.6E-01	rs997263	2.0E-01	7.1E-01
11	*NCR3LG1*	17 373 279	17 398 868	13	rs6486364	4.9E-02	1.9E-01	rs12791318	1.1E-01	3.7E-01
11	*IL18*	112 013 976	112 034 840	17	rs1834481	1.0E-01	3.9E-01	rs2043055	1.5E-01	5.1E-01
11	*CXCR5*	118 754 541	118 766 971	14	rs12363277	1.0E-01	3.6E-01	rs12363277	5.1E-02	1.9E-01
12	*KLRK1*	10 524 952	10 542 640	20	rs2617149	1.5E-02	8.2E-02	rs12826560	9.0E-02	3.7E-01

12	*KLRC4*	10 559 983	10 562 356	5	rs2734565	1.4E-01	2.9E-01	rs2617170	4.2E-01	7.2E-01
12	*KIF21A*	39 687 030	39 836 918	71	rs11171691	2.4E-02	2.6E-01	rs11172108	4.9E-02	4.3E-01
12	*BIN2*	51 674 822	51 717 938	20	rs4761998	6.4E-02	2.9E-01	rs4761995	5.9E-02	2.6E-01
12	*IFNG*	68 548 550	68 553 521	4	rs1861494	6.6E-01	8.9E-01	rs1861493	3.4E-01	5.8E-01
12	*CMKLR1*	108 681 821	108 733 094	48	rs11113818	8.2E-02	5.1E-01	rs10861889	5.1E-02	3.6E-01
13	*FREM2*	39 261 173	39 461 268	220	rs11618650	4.1E-03	1.5E-01	rs2218722	1.3E-02	3.3E-01
13	*PCDH20*	61 983 818	61 989 655	6	rs3829388	1.8E-01	3.9E-01	rs3812872	3.0E-01	6.1E-01
15	*CD276*	73 976 622	74 006 859	35	rs12591553	3.1E-01	8.9E-01	rs12594595	9.0E-02	4.7E-01
16	*ZNF434*	3 432 085	3 451 025	14	rs28603	8.1E-02	2.9E-01	rs17136367	2.3E-01	6.4E-01
16	*ZNF174*	3 451 190	3 459 364	5	rs39728	3.7E-01	6.6E-01	rs37811	2.1E-01	4.3E-01
16	*ITGAX*	31 366 509	31 394 318	16	rs2929	1.9E-01	6.0E-01	rs8052139	4.7E-02	1.9E-01
17	*CCL13*	32 683 471	32 685 629	2	rs159313	7.2E-01	8.5E-01	rs2072069	8.5E-01	9.4E-01
17	*CCL1*	32 687 399	32 690 252	5	rs3136682	4.4E-02	1.0E-01	rs3136682	2.3E-01	4.5E-01
17	*CCL16*	34 303 535	34 308 523	4	rs2063979	8.2E-01	9.7E-01	rs11080369	3.4E-02	6.6E-02
17	*CCL4*	34 431 220	34 433 014	2	rs1719147	4.4E-02	6.0E-02	rs1634517	5.0E-01	6.3E-01
17	*ZNHIT3*	34 842 473	34 851 662	4	rs2306589	8.1E-02	1.6E-01	rs2277662	2.4E-01	4.3E-01
18	*SERPINB3*	61 322 431	61 329 197	3	rs1065205	4.5E-01	6.7E-01	rs7228687	2.0E-01	3.3E-01
19	*ELAVL1*	8 023 457	8 070 529	24	rs7251814	3.9E-01	9.1E-01	rs12977189	8.3E-02	3.8E-01
19	*CCL25*	8 117 934	8 127 547	8	rs2287936	3.9E-01	7.7E-01	rs2032887	3.5E-01	7.2E-01
19	*C19orf66*	10 196 806	10 203 928	2	rs2232066	3.9E-02	5.3E-02	rs2232066	9.5E-02	1.2E-01
19	*CDKN2D*	10 677 138	10 679 655	1	rs1465701	6.3E-01	6.1E-01	rs1465701	2.7E-01	2.4E-01
19	*ZNF20*	12 242 803	12 251 140	2	rs155955	2.1E-01	2.8E-01	rs12608894	1.9E-01	2.5E-01
19	*RCN3*	50 030 875	50 046 890	5	rs10419198	3.3E-01	6.1E-01	rs8108243	1.9E-01	3.9E-01
20	*SIRPD*	1 514 897	1 538 343	26	rs16995146	5.1E-02	2.7E-01	rs2249673	2.3E-01	7.5E-01
21	*APP*	27 252 861	27 543 446	306	rs7281055	8.2E-03	3.2E-01	rs2830076	3.1E-02	6.8E-01
21	*COL18A1*	46 825 097	46 933 634	82	rs2236483	1.3E-01	7.8E-01	rs17004785	2.0E-01	9.1E-01
22	*TPST2*	26 921 714	26 986 089	94	rs5752349	3.4E-02	4.0E-01	rs4149484	4.7E-02	4.8E-01

Start and end positions of each gene are in accordance with Build 37.1.
The genes at known asthma loci are in bold.

Supplementary Methods 1: computing gene-level *P*-values via fastCGP. We take advantage of the Circular Genomic Permutation (CGP) strategy[4] and propose an efficient and exact method, named fastCGP, to compute gene-level *P*-values from SNP-level *P*-values of a GWAS. CGP is a randomization method that permutes SNP-level statistics in a genomic manner to preserve the genomic structure such as regional linkage disequilibrium (LD), thereby to keep similar patterns of correlation in the permutated data as in the original data. Briefly, it considers the genome to be circular and ordered from chromosome 1 to chromosome 22. SNP-level *P*-values of a GWAS are ordered according to the position of the SNPs on the circle. A CGP sample is generated by rotating the ordered statistics for a random position and reassigning them to each SNP. This randomization strategy has been successfully applied to several studies[5–7], and was shown to have similar performances compared to the gold standard of phenotype permutation in the context of pathway analysis[8].

Our method starts by mapping SNPs to genes (between the start site and 3'-untranslated region of each gene) using dbSNP Build 132 and human Genome Build 37.1 (a user can choose another mapping strategy). The gene-level *P*-value of a gene g, denoted as P_g, is represented by the best SNP *P*-value among all SNPs mapped to the gene. This *P*-value is biased by gene length (amount of mapped SNPs) as genes with more SNPs mapped tend to have a lower best SNP *P*-value by chance. We correct for such bias using a permutation test framework. We define the corrected *P*-value as $P_{\text{corrected}} = 1 - l / (L+1)$, where L is the total number of CGP samples; l is the number of samples with $P_{\pi,g} > P_g$, which we call as *normal CGP samples* (nCGP). Particularly, we include all non-repeating CGP samples so that to obtain the best obtainable *P*-value within this permutation test framework. In this case, L becomes the total amount of SNPs placed on the circle (hence is the number of SNPs in a GWAS), while l can be calculated analytically without generating any CGP sample. For illustration convenience, we call a SNP *P*-value as an extreme *P*-values if it is less than or equal to P_g. We say two extreme *P*-values are consecutive if there is no other extreme *P*-value placed between them on the circle. Note that the SNPs mapped to a gene are consecutive on the circle, hence by rotating the SNP *P*-values, it generates a nCGP only if all *P*-values reassigned to gene g are located between some pair of consecutive extreme *P*-values, say, $P_i \sim P_j$ ($1 \le i, j \le L$ are the SNP positions on the circle). Denote d_{ij} as the number of positions between P_i and P_j on the circle, m as the number of SNPs assigned to the *gene*, $I_{(\cdot)}$ as the indicator function. Then the number of unique rotations with all reassigned *P*-values located within $P_i \sim P_j$ is equal to $\gamma_{ij} = (d_{ij} - m + 1) I_{(d_{ij} \ge m)}$. Since the total amount of non-repeating nCGPs is the summation of γ_{ij} for all pairs of consecutive extreme *P*-

values, this leads to the formula $P_{\text{corrected}} = 1 - \sum_{i\sim j} \gamma_{ij} / (L+1)$. The complete algorithm is summarized below

Algorithm Computation of corrected gene-level *P*-values via fastCGP

**Step 1.**	Order GWAS SNP *P*-values on a circle according to the genomic positions of SNPs
**Step 2.**	Map SNPs to genes according to their genomic positions
**Step 3.**	For a gene g , set P_g as the minimum *P*-value of all SNPs mapped to it
**Step 4.**	Find all extreme SNP *P*-values on the circle: $\{P_{SNP} \mid P_{SNP} \le P_g\}$
**Step 5.**	Compute $\gamma_{ij} = (d_{ij} - m + 1) I_{(d_{ij} \ge m)}$ for all pairs of consecutive extreme *P*-value $P_i \sim P_j$
**Step 6.**	Compute the corrected gene-level *P*-value: $P_{\text{corrected}} = 1 - \sum_{i\sim j} \gamma_{ij} / (L+1)$

In the following we present an illustrative example of fastCGP. We constructed an artificial GWAS result consisting of $L = 100$ SNP *P*-values (Supplementary Fig. S5). We set all *P*-values as 0.1 except $P_{11} = 0.05, P_{12} = 0.02, P_{14} = 0.04$, and $P_{18} = 0.07$. These *P*-values are ordered on a circle according to the chromosomal position of corresponding SNPs. A gene g has $m = 3$ SNPs mapped to its genomic region. Its uncorrected *P*-value P_g is set as the minimum SNP *P*-value among the three mapped SNPs ($P_g = P_{18} = 0.07$). There are four extreme SNP *P*-values on the circle : P_{11}, P_{12}, P_{14} and P_{18}. The consecutive extreme *P*-value pairs are $P_{11} \sim P_{12}, P_{12} \sim P_{14}, P_{14} \sim P_{18}, P_{18} \sim P_{11}$. For each pair, the amount of unique rotations with all SNP *P*-values reassigned to gene g falling into this pair is 0, 0, 1 and 92 respectively. Thereby $P_{corrected} = 1 - (0 + 0 + 1 + 92)/101 = 0.079$.

We compared the performance of fastCGP with two other popular methods VEGAS2[2] and MAGMA[3]. The META2 asthma dataset in our study was used for the comparison. For both VEGAS2 and MAGMA we implemented their best-SNP sub-model (use *-bestsnp* option for VEGAS2 and *snp-wise=top,1* option for MAGMA). Both methods apply Monte-Carlo simulations to correct the best-SNP *P*-value for the gene length bias. During simulation, the LD patterns between SNPs within a gene are estimated on the basis of the LD structure of a set of reference individuals. As we could not use the original genotypes of our asthma dataset, we used the 1,000 Genomes European population as external reference. We observed that the results obtained by fastCGP were concordant with those obtained using VEGAS2 or MAGMA. At the chromosome level, the Pearson correlation coefficients between the gene-level *P*-values (-log10 transformed) of fastCGP and VEGAS2 range from 0.93 to 0.97, with an average value of 0.96 (Supplementary Fig. S6). The correlation coefficients of gene-level *P*-values between fastCGP and MAGMA range from 0.95 to 0.98, with an average value of 0.97 (Supplementary Fig. S7). As

for computational efficiency, both fastCGP and MAGMA took ~30 minutes on a PC (Intel Core i7 3.40GHz CPU, 8GB RAM); while VEGAS2 took 13 hours to perform the analysis.

Supplementary Methods 2: generating random modules via Metropolis-Hasting Random Walk algorithm. We inherited the Metropolis-Hasting Random Walk (MHRW) algorithm[9] to generate random modules. It has the property that the stationary probability of each node to be sampled follows the uniform distribution, and has been demonstrated to work well in practice[9]. In the beginning, a seed gene V is chosen from the scored-PPI. The next gene W is selected at random from all neighbours of V. W is added to the module and set as the next seed if the degree ratio of V and W is smaller than a random number drawn from uniform distribution $U(0,1)$. Otherwise stay at V and repeat the step. The procedure iterates until the module has the same number of genes as the module under test. To ensure sufficient coverage of the whole scored-PPI, we set each gene in the network as a seed to generate a module. Then, a total of N (equals to the number of genes in the scored-PPI) random modules are generated.

References

1. Moffatt, M. F. *et al.* A large-scale, consortium-based genomewide association study of asthma. *N. Engl. J. Med.* **363,** 1211–1221 (2010).
2. Mishra, A. & Macgregor, S. VEGAS2: Software for More Flexible Gene-Based Testing. *Twin Res. Hum. Genet.* **18,** 86–91 (2015).
3. Leeuw, C. A. de, Mooij, J. M., Heskes, T. & Posthuma, D. MAGMA: Generalized Gene-Set Analysis of GWAS Data. *PLOS Comput. Biol.* **11,** e1004219 (2015).
4. Cabrera, C. P. *et al.* Uncovering networks from genome-wide association studies via circular genomic permutation. *G3 Bethesda Md* **2,** 1067–1075 (2012).
5. Stainton, J. J. *et al.* Detecting signatures of selection in nine distinct lines of broiler chickens. *Anim. Genet.* **46,** 37–49 (2015).
6. Mott, R. *et al.* The architecture of parent-of-origin effects in mice. *Cell* **156,** 332–342 (2014).

7. Chambers, E. V., Bickmore, W. A. & Semple, C. A. Divergence of mammalian higher order chromatin structure is associated with developmental loci. *PLoS Comput. Biol.* **9,** e1003017 (2013).
8. Brossard, M. *et al.* Comparison of permutations strategies to assess gene-set significance in gene-set-enrichment analysis. *22nd annual meeting of the International Genetic Epidemiology Society (IGES), 15-17 Sept. 2013, Chicago (USA). Abstract published online at www.geneticepi.org/meeting-abstracts/.*
9. Gjoka, M., Kurant, M., Butts, C. T. & Markopoulou, A. Walking in Facebook: A Case Study of Unbiased Sampling of OSNs. in *2010 Proceedings IEEE INFOCOM* 1–9 (2010). doi:10.1109/INFCOM.2010.5462078
